# Serum KL-6 levels reflect the severity of interstitial lung disease associated with connective tissue disease

**DOI:** 10.1186/s13075-019-1835-9

**Published:** 2019-02-14

**Authors:** Jeong Seok Lee, Eun Young Lee, You-Jung Ha, Eun Ha Kang, Yun Jong Lee, Yeong Wook Song

**Affiliations:** 1 Division of Rheumatology, Department of Internal Medicine, Seoul National University College of Medicine, 101, Daehak-ro, Jongno-gu, Seoul, 03080 South Korea; 2 Division of Rheumatology, Department of Internal Medicine, Seoul National University Bundang Hospital, Gyeonggi-do, South Korea

**Keywords:** Connective tissue disease, KL-6, Interstitial lung disease, Pulmonary function test

## Abstract

**Background:**

Biomarkers have been actively investigated to supplement functional and imaging modalities to predict the severity, therapeutic responsiveness, and progression of connective tissue disease-associated interstitial lung disease (CTD-ILD). This study aimed to evaluate Krebs von den Lungen 6 (KL-6) as a potential biomarker reflecting the severity of CTD-ILD as assessed through computed tomography (CT) and pulmonary function test (PFT) parameters.

**Methods:**

This retrospective study included 549 Korean patients with rheumatoid arthritis, systemic sclerosis, inflammatory myositis, and other CTDs with or without concurrent ILD. Serum KL-6 concentration (U/mL) was measured using the latex-enhanced immunoturbidimetric assay method. CT and PFT results were collected within 1 year of serum collection. A semiquantitative grade of ILD extent was evaluated through CT scan (grade 1, 0–25%; grade 2, 26–50%; grade 3, 51–75%; grade 4, 76–100%).

**Results:**

CTD-ILD patients (*n* = 165) had elevated serum KL-6 levels compared to CTD patients without ILD (*n* = 384) (*p* < 0.001), and those findings were preserved after adjusting for age, sex, and CTD type. The semiquantitative grade of ILD on CT scan was significantly proportional to the KL-6 level, and the optimal cut-off KL-6 value effectively differentiated each ILD grade. The percent diffusing capacity of the lung for carbon monoxide (DLCO) (*p* < 0.001) and forced vital capacity (FVC) (*p* < 0.001) parameters had a moderate, negative correlation with the KL-6 level.

**Conclusion:**

Serum KL-6 levels were increased in CTD-ILD patients and had a positive correlation with CT grade and a negative correlation with FVC and DLCO. Serum KL-6 levels may reflect CTD-ILD severity.

**Electronic supplementary material:**

The online version of this article (10.1186/s13075-019-1835-9) contains supplementary material, which is available to authorized users.

## Background

Interstitial lung disease (ILD) is commonly associated with various connective tissue diseases (CTDs), such as rheumatoid arthritis (RA), systemic sclerosis (SSc), inflammatory myositis (IM), Sjogren’s syndrome (SS), and systemic lupus erythematosus (SLE) [1]. Compared to idiopathic pulmonary fibrosis, CTD-ILD has a relatively less severe prognosis [2]. However, depending on the type of ILD pathology and underlying CTD, the clinical outcome of CTD-ILD is different, and timely treatment should be considered in certain conditions for survival [2–5]. In the case of SSc, the role of cyclophosphamide for the treatment of ILD was established by well-designed clinical trials [6], but deciding when and how long to treat patients with cyclophosphamide is still complex in clinical practice [7]. Mycophenolate mofetil has also shown therapeutic efficacy in SSc-associated ILD [8, 9]. In cases of rapidly progressing IM-associated ILD, combination therapy with high-dose corticosteroids and immunosuppressive agents should be considered as an early intervention [10, 11]. Therefore, sensitive detection and sophisticated evaluation of CTD-ILD could be critical to improve the clinical outcome of these patients.

Currently, high-resolution computed tomography (HRCT) and pulmonary function test (PFT) are the two major modalities used to diagnose and evaluate CTD-ILD rather than lung biopsy [12]. The typical PFT battery for CTD-ILD comprises a restrictive ventilation and decreased diffusing capacity of the lung for carbon monoxide (DLCO). However, when other pulmonary conditions, such as emphysema or pulmonary hypertension, are combined with ILD, they confound the interpretation of PFT results [13]. Furthermore, respiratory failure due to acute exacerbation of ILD often inhibits patients from properly performing PFT. Currently, the pathological pattern of parenchymal lesions of CTD-ILD can be estimated through HRCT. In general, nonspecific interstitial pneumonia is the most common histological type among CTD-ILDs, but patterns such as usual interstitial pneumonia, bronchiolitis obliterans organizing pneumonia, and lymphocytic interstitial pneumonia may exist alone or together in a substantial number of cases [14]. However, the corresponding type of CTD-ILD suggested by HRCT is insufficient to evaluate the current disease status or predict disease progression [15]. Specific features detected on HRCT, such as ground-glass opacities, are usually regarded as a sign of early and active disease status, which can be improved by treatment, although recent studies have disputed this view [16, 17]. Nevertheless, HRCT is considered a standard for evaluating CTD-ILD.

Biomarkers have been actively investigated to supplement these functional (i.e., PFT) and imaging (i.e., HRCT) modalities to predict the severity, therapeutic responsiveness, and progression of CTD-ILD. In general, biomarkers of ILD can be classified according to their origin based on whether they are associated with lung epithelium or not [18]. Among the lung epithelium-specific proteins, sialylated glycoprotein Krebs von den Lungen-6 (KL-6) has been investigated as an important biomarker that is directly associated with the pathogenic process of ILD, reflecting the extent of damage and regeneration of type II pneumocytes [19]. Recently, elevated serum KL-6 levels were suggested as an indicator of severity measured through PFT and as a predictor of early progression in patients with SSc [20–22]. The poor prognosis of ILD associated with inflammatory myositis also has a positive correlation with serum KL-6 levels [23, 24]. Although the clinical significance of KL-6 is emerging in the CTD-ILD field, its role in the diagnosis and measurement of ILD requires clarification in patients with various autoimmune diseases that are potentially accompanied by ILD.

The purpose of this retrospective cohort study was to investigate the cross-sectional, quantitative correlation of serum KL-6 levels with the diagnosis and severity of ILD measured by chest CT or PFT, which are the current, standard methods of evaluating CTD-ILD. The association between the serum KL-6 level and progression of ILD was also studied in a subgroup of patients who had follow-up CT and PFT.

## Methods

### Study population

This retrospective study involved 549 Korean patients who were diagnosed with RA (*n* = 147), SSc (*n* = 74), IM (*n* = 108), and other autoimmune diseases (*n* = 220), including SS (*n* = 152) and SLE (*n* = 68), in Seoul National University Hospital between December 2004 and December 2017. A total of 165 patients with ILD were included. Patients with overlapping syndromes or multiple autoimmune diseases were excluded. Clinical information was obtained from the electronic medical record database of Seoul National University Hospital. This study was performed in compliance with the Declaration of Helsinki and was approved by the Institutional Review Board of Seoul National University Hospital (IRB#:0408-131-010). All patients provided written informed consent.

### Measurement of KL-6

Serum KL-6 concentration (U/mL) was measured through the Nanopia KL-6 assay (SEKISUI MEDICAL, Tokyo) using the latex-enhanced immunoturbidimetric assay method.

### Clinical evaluation for ILD

The most recent chest HRCT and PFT results within 12 months of each serum sampling were analyzed. One trained radiologist who was blinded to the clinical information graded the ILD extent of CT scans semiquantitatively (grade 1, 0–25%; grade 2, 26–50%; grade 3, 51–75%; grade 4, 76–100%). If any definitive reason for exacerbation of CT finding other than ILD was present, such as pneumonia, those CT scans were excluded from the analysis. DLCO% and FVC% were calculated from the PFT results. Inflammatory markers such as erythrocyte sedimentation rate (ESR) and C-reactive protein (CRP) were evaluated within 30 days of serum collection. To predict ILD progression, progression of ILD was defined as an increase in semiquantitative CT grade.

### Statistical analysis

To evaluate the differences between patients with and without ILD, baseline statistics (mean, standard deviation, or frequency) of each variable were generated, and their differences were evaluated using Student’s *t* test or chi-squared test. Receiver operating characteristic (ROC) curve analysis was conducted to demonstrate the optimal cut-off value of serum KL-6 to detect or delineate each semiquantitative grade of ILD on the chest HRCT. The area under the curve (AUC) with a 95% confidence interval (CI) and the sensitivity and specificity of the suggested cut-off KL-6 value were calculated. The Pearson correlation coefficient was used to analyze the association between serum KL-6 levels and PFT parameters. *p* values < 0.05 were considered significant. SAS, version 9.1.3 (SAS Institute, Cary, NC), was used for the statistical analysis.

## Results

### Clinical characteristics of the study population

Patients with CTD-ILD (*n* = 165) had elevated serum KL-6 levels compared to CTD patients without ILD (*n* = 384) (*p* < 0.001) (Table 1). The mean age (*p* < 0.001) and mean value of ESR (*p* = 0.006) were significantly higher in the ILD group. After matching age, sex, and type of CTD of both groups, we conducted a subgroup analysis with 129 patients from each group. The results showed that the serum KL-6 level was consistently higher in the ILD group (*p* < 0.001). The difference in mean ESR was present after matching, and it was still higher in the ILD group (*p* = 0.004).

**Table 1:** Demographic and clinical characteristics of study participants (*n* = 549) and CTD-matched participants (*n* = 258) stratified by ILD

	Total study population (*n* = 549)			Matched subgroup* (*n* = 258)		
	ILD(+) (*n* = 165)	ILD(−) (*n* = 384)	*p* value	ILD(+) (*n* = 129)	ILD(−) (*n* = 129)	*p* value
Mean age, years (SD)	56.4 (13.0)	51.1 (14.3)	< 0.001	55.1 (14.0)	54.5 (14.8)	0.721
Sex, male (%)	27 (16.4)	44 (11.5)	0.116	25 (19.4)	22 (17.1)	0.628
Type of CTD (%)						
RA	41 (24.8)	106 (27.6)	–	41 (31.8)	41 (31.8)	–
SSc	53 (32.1)	21 (5.5)	–	21 (16.3)	21 (16.3)	–
IM	56 (33.9)	52 (13.5)	–	52 (40.3)	52 (40.3)	–
Others (SLE or SS)	15 (9.2)	205 (53.4)	–	15 (11.6)	15 (11.6)	–
Inflammatory markers† (SD)						
Mean ESR, mm/h	39.0 (24.9)	32.4 (25.7)	0.006	39.5 (24.5)	30.2 (26.3)	0.004
Mean CRP, mg/dL	0.97 (1.98)	1.01 (1.97)	0.828	1.12 (2.13)	1.03 (1.97)	0.744
Serum KL-6, U/mL (SD)	741.0 (724.3)	236.1 (157.0)	< 0.001	693.8 (679.0)	256.0 (224.4)	< 0.001
Pulmonary function (SD)						
Mean FVC%	77.2 (17.3)	–	–	77.5 (17.3)	–	–
Mean DLCO%	61.3 (16.9)	–	–	63.2 (16.4)	–	–
Semiquantitative CT grade (%)	(*n* = 145)			(*n* = 112)		
Grade 1 (0–25)	61 (42.1)	–	–	51 (45.5)	–	–
Grade 2 (26–50)	39 (26.9)	–	–	31 (27.7)	–	–
Grade 3 (51–75)	31 (21.4)	–	–	20 (17.9)	–	–
Grade 4 (76–100)	14 (9.6)	–	–	10 (8.9)	–	–

*Age, sex, and type of CTD were matched between ILD(+) and ILD(−) groups

†At the nearest date within 30 days

CRP, C-reactive protein; CT, computed tomography; CTD, connective tissue disease; ESR, erythrocyte sedimentation rate; DLCO%, diffusing capacity of carbon monoxide % predicted; FVC%, forced vital capacity % predicted; ILD(+), presence of interstitial lung disease; ILD(−), absence of interstitial lung disease; IM, inflammatory myositis; PFT, pulmonary function test; RA, rheumatoid arthritis; SD, standard deviation; SS, Sjogren’s syndrome; SLE, systemic lupus erythematosus; SSc, systemic sclerosis

**Fig. 1:**
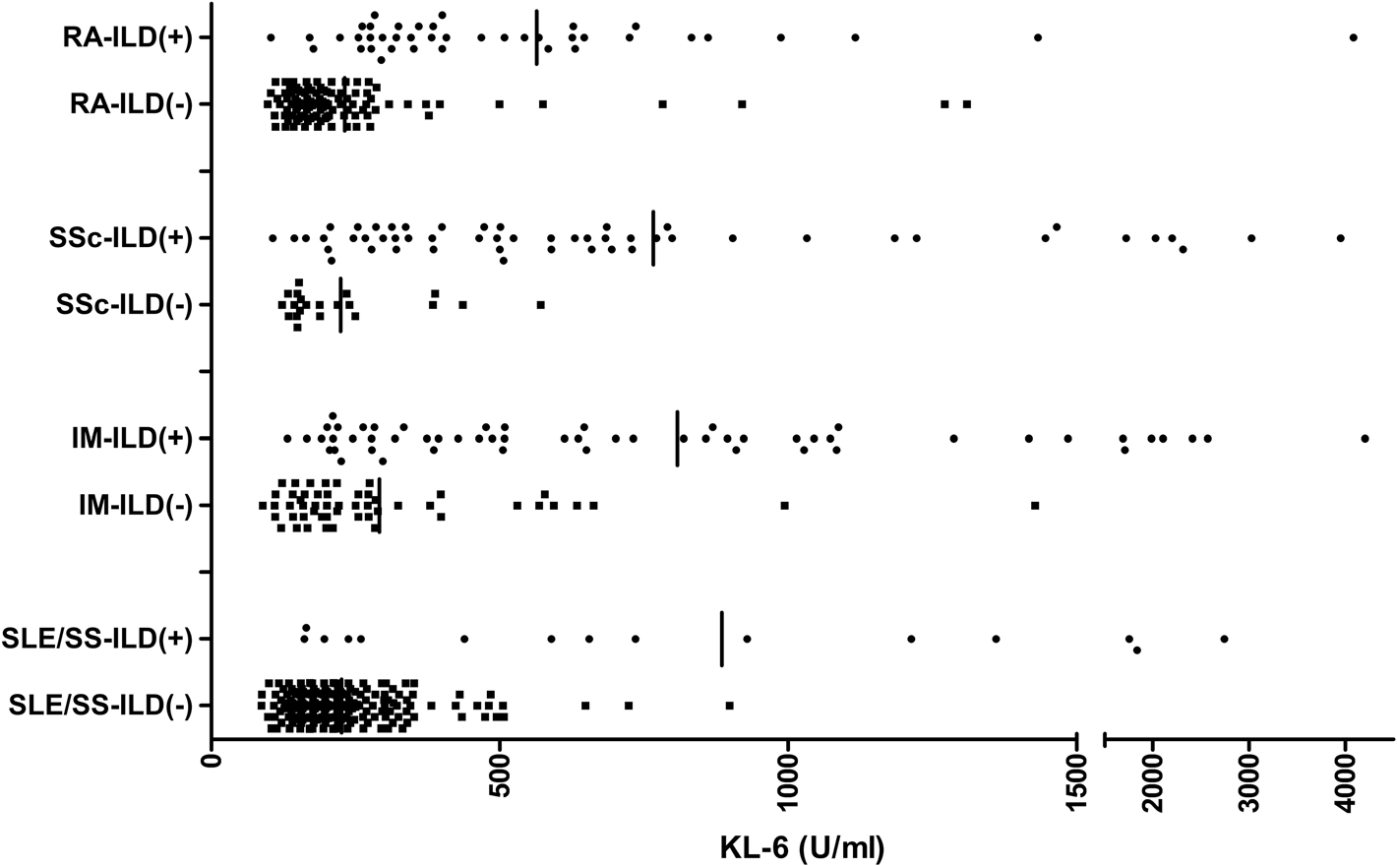
Serum KL-6 level of the study population according to the presence of ILD in each CTD. CTD, connective tissue disease; ILD(+), presence of interstitial lung disease; ILD(−), absence of interstitial lung disease; IM, inflammatory myositis; RA, rheumatoid arthritis; SS, Sjogren’s syndrome; SLE, systemic lupus erythematosus; SSc, systemic sclerosis

When we stratified by the type of underlying CTD, including patients with RA [mean (SD) 563.9 (627.0) vs. 231.3 (188.5) U/mL, *p* < 0.001], SSc [766.4 (754.6) vs. 224.0 (188.5) U/mL, *p* = 0.002], IM [808.1 (746.9) vs. 291.4 (238.6) U/mL, *p* < 0.001], and others (SS or SLE) [884.9 (762.3) vs. 225.7 (107.0), *p* < 0.001], significantly higher serum KL-6 values were observed in patients with ILD than in patients without ILD (Fig. 1). Although the patients who had ILD with SLE, SS, IM, or SSc tended to have relatively higher serum KL-6 values than the values in those who had ILD with RA, the differences in serum KL-6 levels among the CTD types were not significant.

### Serum KL-6 and severity of ILD

**Fig. 2:**
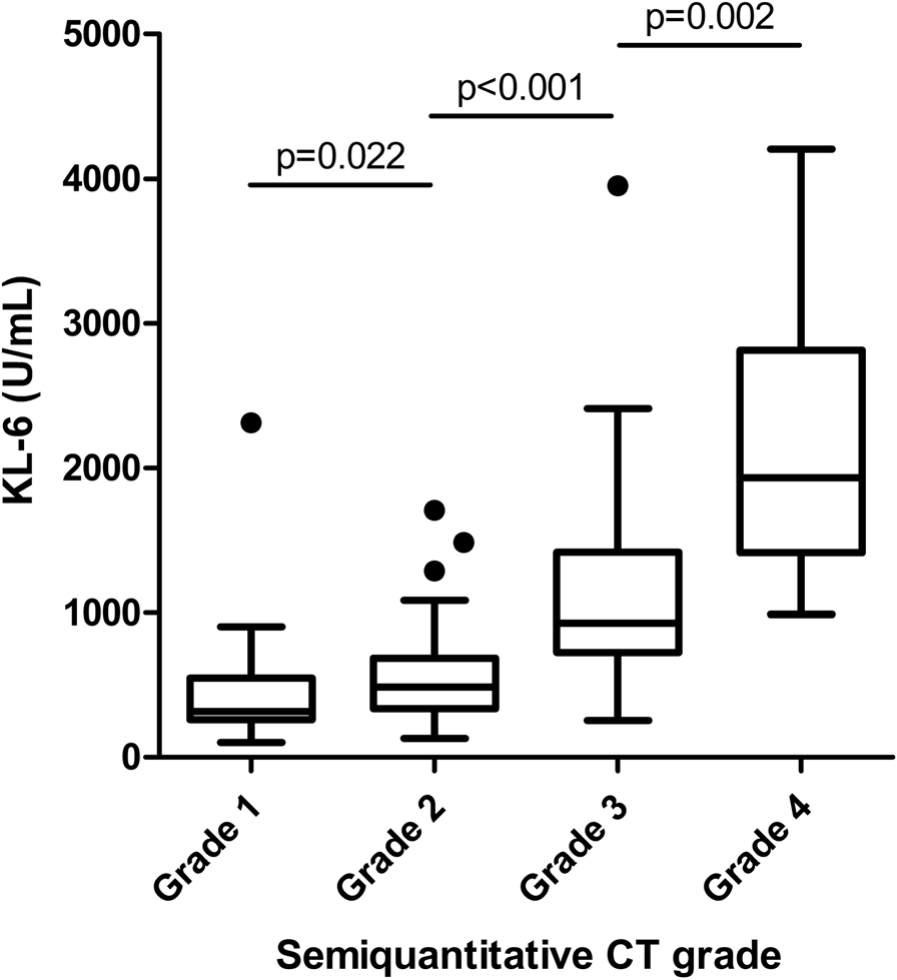
Serum KL-6 levels of ILD patients according to semiquantitative CT grades. CT, computed tomography; grade 1, 0–25% involvement of ILD on chest CT; grade 2, 26–50%; grade 3, 51–75%; grade 4, 76–100%

Semiquantitative grades of ILD on the CT scan were significantly proportional to the KL-6 level (Fig. 2). Serum KL-6 levels successfully differentiated grades 1 and 2 (*p* = 0.022), grades 2 and 3 (*p* < 0.001), and grades 3 and 4 (*p* = 0.002) in patients with CTD-ILD. Therefore, serum KL-6 can be used to reflect the current status of CTD-ILD defined by CT scans.

To utilize the serum KL-6 level in clinical practice, the cut-off KL-6 values were suggested to reflect the status measured using CT scans. ROC analysis showed 275.1 U/mL as the serum KL-6 concentration for detecting the presence of ILD in CTD patients (sensitivity 79.4%, specificity 79.9%) (Additional file 1: Figure S1). Then, semiquantitative grades on CT scans were divided depending on the serum KL-6 levels (Table 2). The AUC values of those KL-6 levels for differentiating each CT grade were consistently larger than the AUC values of FVC% and DLCO%.

**Table 2:** AUC, optimal cut-off value, and sensitivity and specificity of serum KL-6 level, FVC%, and DLCO% for semiquantitative CT grade differentiation of ILD using a ROC curve

	Semiquantitative CT grade		
	Grade 1 vs Grades 2, 3, 4	Grades 1, 2 vs Grades 3, 4	Grades 1, 2, 3 vs Grade 4
KL-6			
AUC (95% CI)	0.807 (0.737–0.878)	0.900 (0.843–0.956)	0.953 (0.917–0.988)
Cut-off value, U/mL	684.3	689.7	958.3
Sensitivity, %	58.3	86.7	100
Specificity, %	91.8	86.0	84.0
FVC%			
AUC (95% CI)	0.702 (0.603–0.800)	0.701 (0.603–0.800)	0.735 (0.573–0.898)
Cut-off value	76.5	76.5	62.5
Sensitivity, %	66.7	59.2	73.6
Specificity, %	67.1	78.6	66.7
DLCO%			
AUC (95% CI)	0.759 (0.670–0.849)	0.790 (0.701–0.880)	0.851 (0.731–0.972)
Cut-off value	53.5	61.5	36.0
Sensitivity, %	90.9	58.1	94.1
Specificity, %	57.4	86.8	70.0

AUC, area under the curve; CT, computed tomography; DLCO%, diffusing capacity of carbon monoxide % predicted; FVC%, forced vital capacity % predicted; ILD, interstitial lung disease; PFT, pulmonary function test; ROC, receiver operating characteristic

**Fig. 3:**
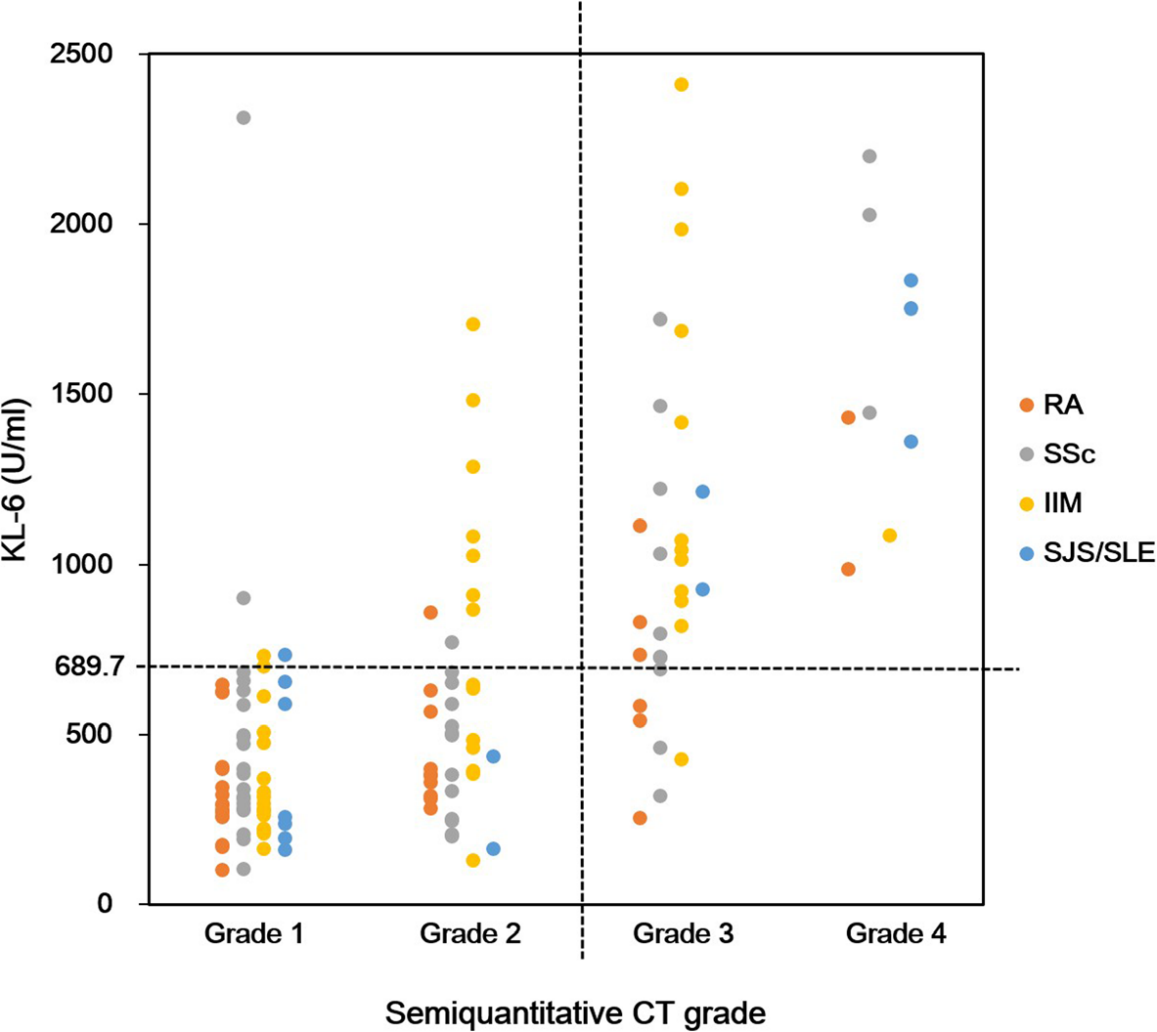
Scatter plot of semiquantitative CT grades and serum KL-6 levels with cut-off value (689.7 U/mL) to discriminate grades 1–2 from grades 3–4

Especially for discriminating grades 3 and 4 from grades 1 and 2, the AUC was 0.900, and the cut-off value of the serum KL-6 level was 689.7 U/mL with high sensitivity (86.7%) and specificity (86.0%). In other words, the CT-measured extent of ILD of more than 50% of patients could be reliably detected by this cut-off serum KL-6 level (Additional file 1: Figure S2). When we applied the cut-off value and analyzed the outliers, such as the patients who had higher KL-6 levels than the cut-off value with CT grades 1–2 (*n* = 14), 12 of the 14 patients had underlying SSc (*n* = 3) or IM (*n* = 9) (Fig. 3). In contrast, among those who had lower serum KL-6 levels than the cut-off serum KL-6 level with CT grades 3–4 (*n* = 6), half of the patients had RA.

**Fig. 4:**
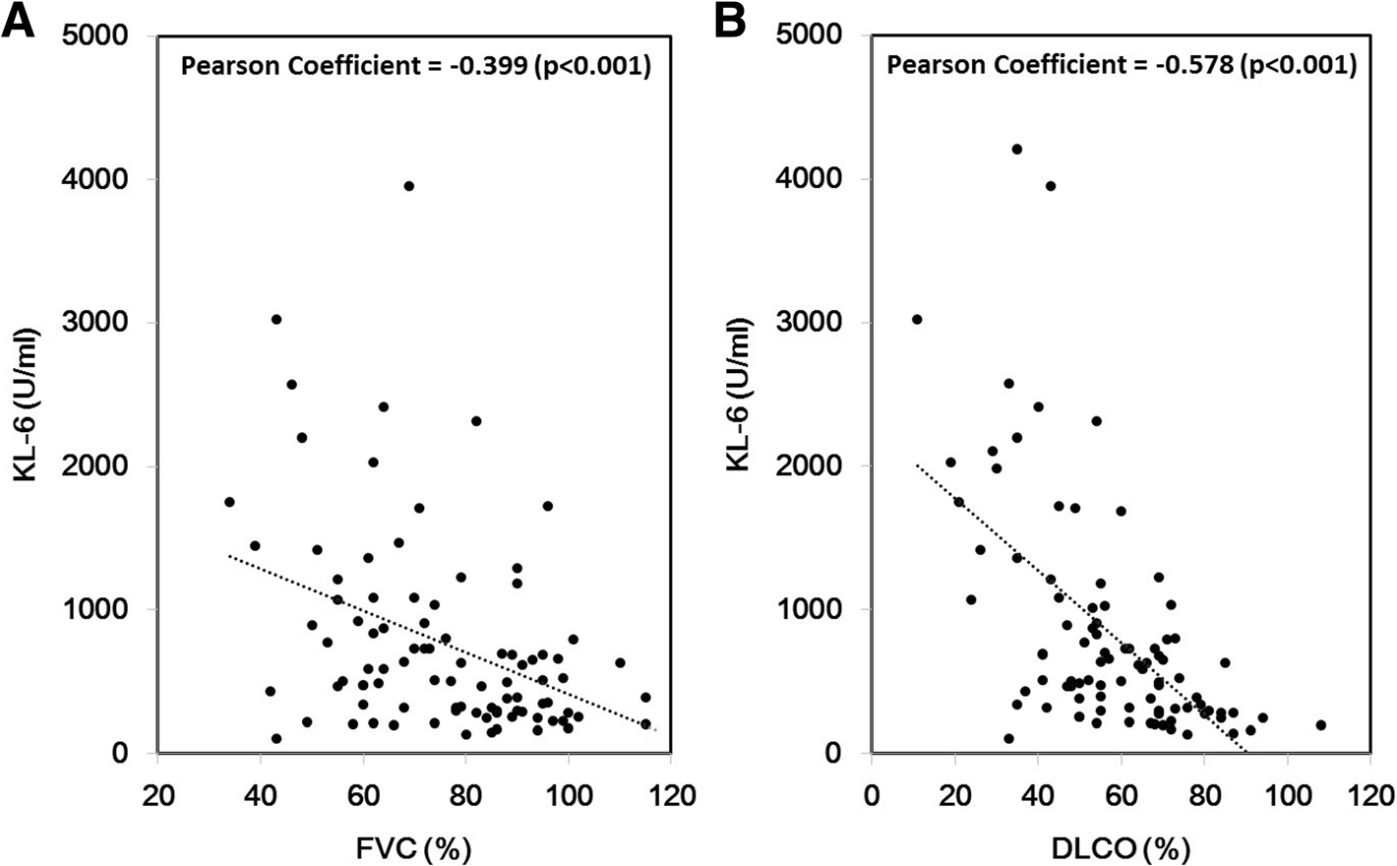
Association between serum KL-6 level and **a** FVC% or **b** DLCO% of patients with ILD. DLCO%, diffusing capacity of carbon monoxide % predicted; FVC%, forced vital capacity % predicted; ILD, interstitial lung disease

Serum KL-6 levels also had a moderate negative correlation with both FVC% (*p* < 0.001) and DLCO% (*p* < 0.001) (Fig. 4).

### Prediction of ILD progression by subgroup analysis

For the subgroup of patients who received a second chest HRCT after serum sampling for KL-6 measurement, we tried to evaluate whether serum KL-6 level at one time point could predict ILD progression defined by CT. However, the differences in serum KL-6 levels between the patients who had ILD with and without progression were not significant (*p* = 0.173) (Additional file 1: Table S1). Serum KL-6 levels tended to be higher in patients with CT progression.

## Discussion

In this study, we retrospectively investigated whether KL-6 could be used as a biomarker that reflects the clinical status of CTD-ILD patients. To our knowledge, this is the first study evaluating KL-6 and included the largest sample size of CTD-ILD patients. We mainly focused on the practical aspect of KL-6 to be used in rheumatology clinics for the detection and follow-up of ILD patients with CTDs. Among the patients diagnosed with CTDs with possible ILD manifestations, the serum KL-6 level was significantly higher in patients with ILD than in those without ILD. Furthermore, the severity of CTD-ILD measured through chest HRCT had a significant, positive correlation with serum KL-6 level, and certain cut-off KL-6 values were suggested for ILD grades. The PFT parameters also had a significant negative correlation with serum KL-6 levels. There may be additional value to measure KL-6 for CTD patients.

First, KL-6 may have a substantial role for evaluating ILD among CTD patients. Evaluation of ILD through regular chest HRCT for patients with SSc or IM can be justified because of the relatively high prevalence and potential life-threatening course in this patient population. However, for other CTDs, such as RA, SS, and SLE, established epidemiological data, including the incidence, prevalence, and outcome of ILDs, are lacking; therefore, regular chest HRCT for patients with these CTDs is not currently recommended. Considering cost-effectiveness and radiation hazard, KL-6 measurement by simple blood test would be a good alternative to chest HRCT for evaluating the current status of ILD in rheumatology clinics regardless of the CTD type. Second, serial measurement of the KL-6 level could be a monitoring method for the exacerbation of ILD. Chest HRCT can provide objective evidence of exacerbation with overt clinical symptoms, but the optimal time interval between CT scans in asymptomatic ILD patients is difficult to determine. Regular, such as yearly, chest HRCT combined with more frequent KL-6 measurements could be an ideal protocol for surveillance of exacerbating CTD-ILD.

Serum KL-6 concentrations lower than 275.1 U/mL were able to differentiate the absence of ILD. In a previous study, 500 U/mL was reported as a cut-off value for diagnosing ILD in cancer patients [25]. Because the KL-6 level may be affected by various lung conditions, such as cancer [26], the appropriate cut-off value for a specific condition needs to be drawn based on a comparison with adequate control. In our study, the corresponding CTD patients without ILD had a mean serum KL-6 level of 256 U/mL, and those with grade 1 ILD had a mean serum KL-6 level of 414 U/mL after matching for age, sex, and type of CTD. More accurate serum KL-6 levels for ILD screening and diagnosis might require a study involving a prospective cohort.

We observed that two patients who had extremely high serum KL-6 levels (over 1000 U/mL) with semiquantitative HRCT grades 1 and 2 eventually experienced rapid progression. However, the subgroup analysis failed to show the predictive ability of KL-6 at one moment. As this subgroup did not have a regular follow-up, a significant level of survival bias could have affected the result. Indeed, KL-6 was able to predict the progression of IM-ILD and prognosis of SSc-ILD in a cohort with more longitudinal data [21, 24]. The utility of KL-6 as a predictive biomarker of ILD exacerbation still needs to be investigated thoroughly in patients using immunosuppressive agents.

This study had a few limitations. First, although we included a large number of CTD-ILD patients, the mean levels of KL-6 were not significantly different according to the type of CTD. ILD patients with RA tended to have a relatively lower KL-6 value than those with IM or SSc did, but this finding was not statistically significant. In addition, most of the outliers with higher serum KL-6 levels than what was estimated for their CT grades had SSc or IM. Prospective and longitudinal cohorts for each type of CTD would enable more accurate comparisons among the CTD types. The second weakness of this study was the method for defining the control group without ILD. Chest HRCT scans were usually routinely conducted in SSc (90.6%) or IM (82.1%) patients within 2 years, and the group without ILD was confirmed by CT in all cases. In contrast, most RA, SLE, or SS patients without ILD were classified mainly through chest X-ray. This difference could affect the detection sensitivity of ILD and skew the epidemiological profile of the study population. Third, serum KL-6 levels still need to be compared with PFT as a screening tool for ILD. Because of the retrospective nature of this study, CTD patients without ILD did not have PFT results in most cases. A prospective study including regular results of serum KL-6 level, PFT, and chest HRCT data would provide a more elaborate model for evaluating CTD-ILD.

## Conclusion

Serum KL-6 levels were increased in patients with CTD-ILD and had a positive correlation with ILD severity as measured using a semiquantitative CT grading scale, whereas serum KL-6 levels had a negative correlation with PFT parameters. Serum KL-6 could be a clinically useful biomarker in screening and evaluating CTD-ILD.

## Additional file

Additional file 1: **Figure S1.** Receiver operating characteristic curve to demonstrate optimal cut-off value of KL-6 to detect presence of ILD in CTD. Figure S2 ROC curves to evaluate the association between semiquantitative CT grade of ILD and the measurement of KL-6, FVC%, and DLCO%. **Table S1.** Prediction of CTD-ILD progression in a subgroup with follow-up data. (DOCX 200 kb)

## Abbreviations

AUC: Area under the curve
CI: Confidence interval
CRP: C-reactive protein
CTDs: Connective tissue diseases
DLCO: Diffusing capacity of the lung for carbon monoxide
ESR: Erythrocyte sedimentation rate
HRCT: High-resolution computed tomography
ILD: Interstitial lung disease
IM: Inflammatory myositis
KL-6: Krebs von den Lungen-6
PFT: Pulmonary function test
RA: Rheumatoid arthritis
ROC: Receiver operating characteristic
SLE: Systemic lupus erythematosus
SS: Sjogren’s syndrome
SSc: Systemic sclerosis
