# Supplementary material for: Serum KL-6 levels reflect the severity of interstitial lung disease associated with connective tissue disease
Source: Arthritis Res Ther. 2019 Feb 14;21:58. doi: 10.1186/s13075-019-1835-9 (PMC6376648; doi:10.1186/s13075-019-1835-9)
Supplement: Supplementary file 1 — Figure S1. Receiver operating characteristic curve to demonstrate optimal cut-off value of KL-6 to detect presence of ILD in CTD. Figure S2 ROC curves to evaluate the association between semiquantitative CT grade of ILD and the measurement of KL-6, FVC%, and DLCO%. Table S1. Prediction of CTD-ILD progression in a subgroup with follow-up data. (DOCX 200 kb) [file 13075_2019_1835_MOESM1_ESM.docx]

**Supplementary materials**

**Table S1**. Prediction of CTD-ILD progression in a subgroup with follow-up data.

|  | ILD patients with follow-up data | | p Value |
| --- | --- | --- | --- |
| Progression defined by CT* | ILD with progression (n=28) | ILD without progression (n=65) |  |
| Mean KL-6, U/ml (SD) | 957.2 (720.2) | 727.4 (773.9) | 0.173 |

*Any increase in semi-quantitative CT grade

**Figure S1**. Receiver operating characteristic curve to demonstrate optimal cutoff value of KL-6 to detect presence of ILD in CTD.


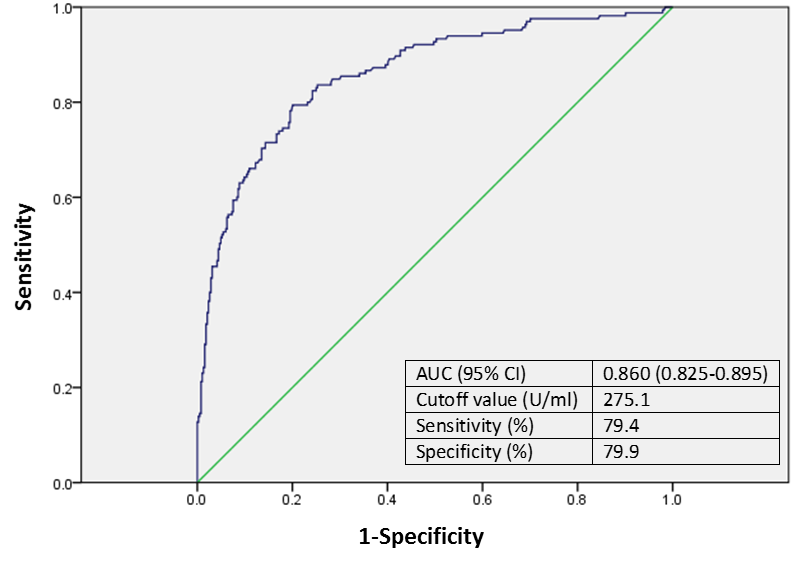


AUC, area under curve; ROC, Receiver operating characteristic; ILD, interstitial lung disease.

**Figure S2**. ROC curves to evaluate the association between semi-quantitative CT grade of ILD and the measurement of KL-6, FVC%, and DLCO%.


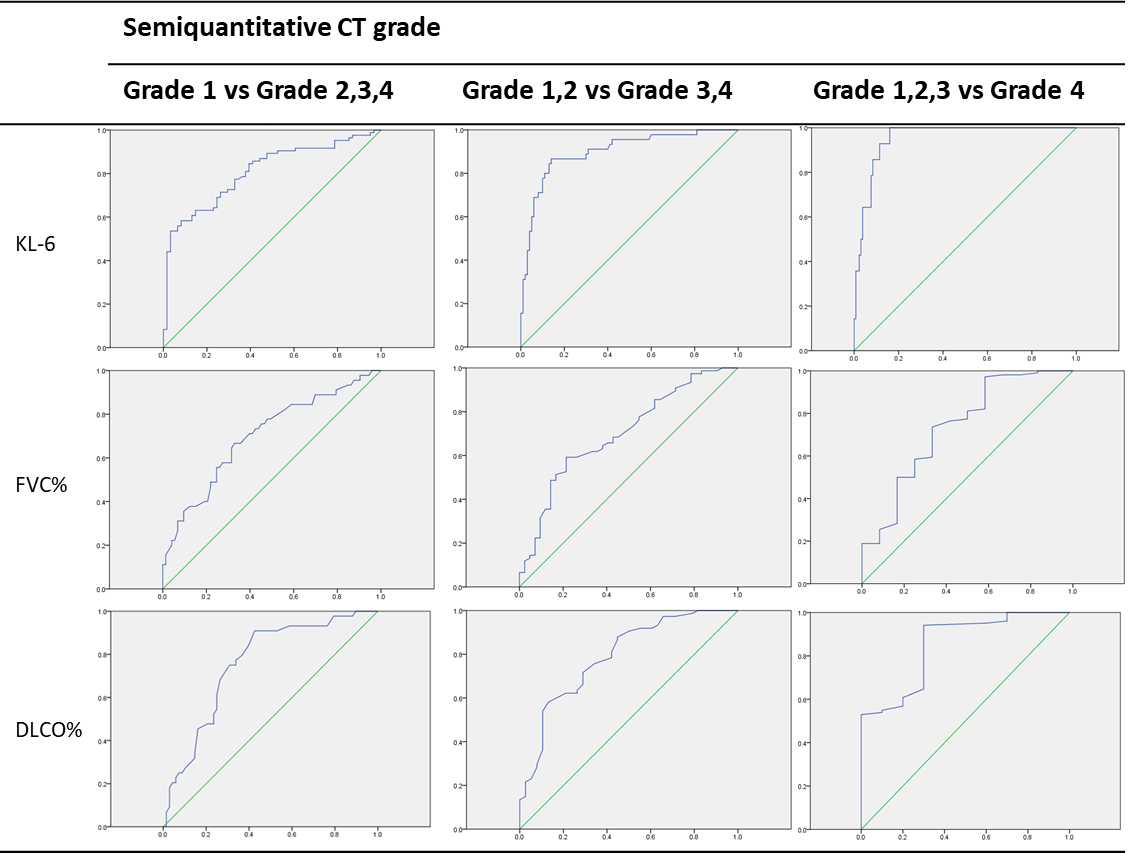


CT, computed tomography; DLCO%, diffusing capacity of carbon monoxide % predicted; FVC%, forced vital capacity % predicted; ILD, interstitial lung disease.
